# Supplementary material for: Single-Cell Profiling Reveals Global Immune Responses During the Progression of Murine Epidermal Neoplasms
Source: Cancers (Basel). 2025 Apr 21;17(8):1379. doi: 10.3390/cancers17081379 (PMC12025564; doi:10.3390/cancers17081379)
Supplement: Supplementary file 1 [file cancers-17-01379-s001.zip › cancers-3549978-supplementary.pdf]

cancers
MDPI

Supplementary Materials

Single-Cell Profiling Reveals Global Immune Responses During the Progression of Murine Epidermal Neoplasms

Xiying Fan, Tonya M. Brunetti, Kelsey Jackson and Dennis R. Roop

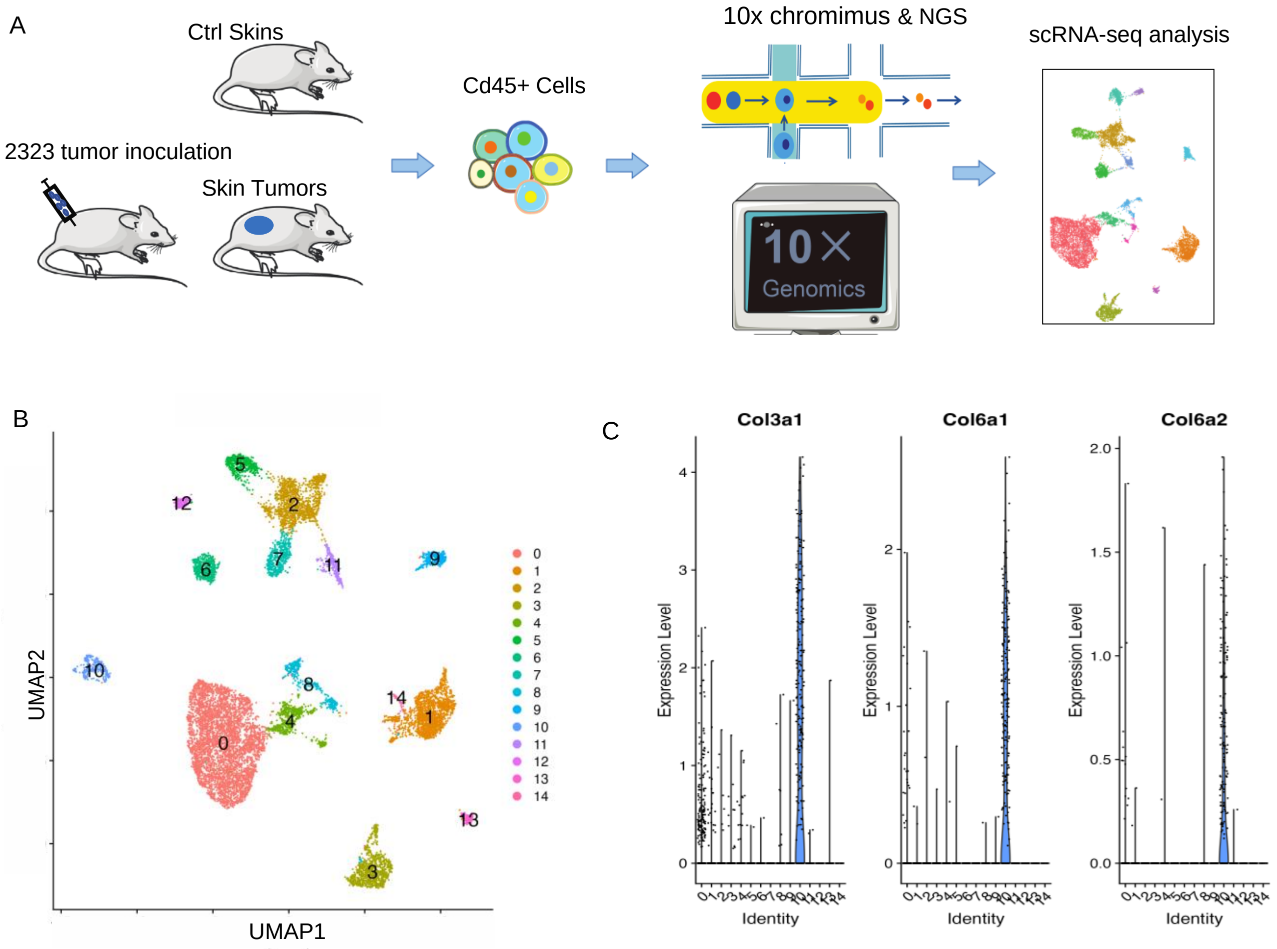

Figure S1. scRNA-seq experimental design. (A) Graphical representation of the experimental setup. Samples were isolated from control skins and skin tumors. Live CD45+ immune cells were FACS-sorted and loaded for scRNA-seq. (B) UMAP projections of 15 immune cell clusters at resolution 0.2. (C) Fibroblast genes Col3a1, Col6a1, Col6a2 genes highly expressed in cluster 10 indicating fibroblast contamination.

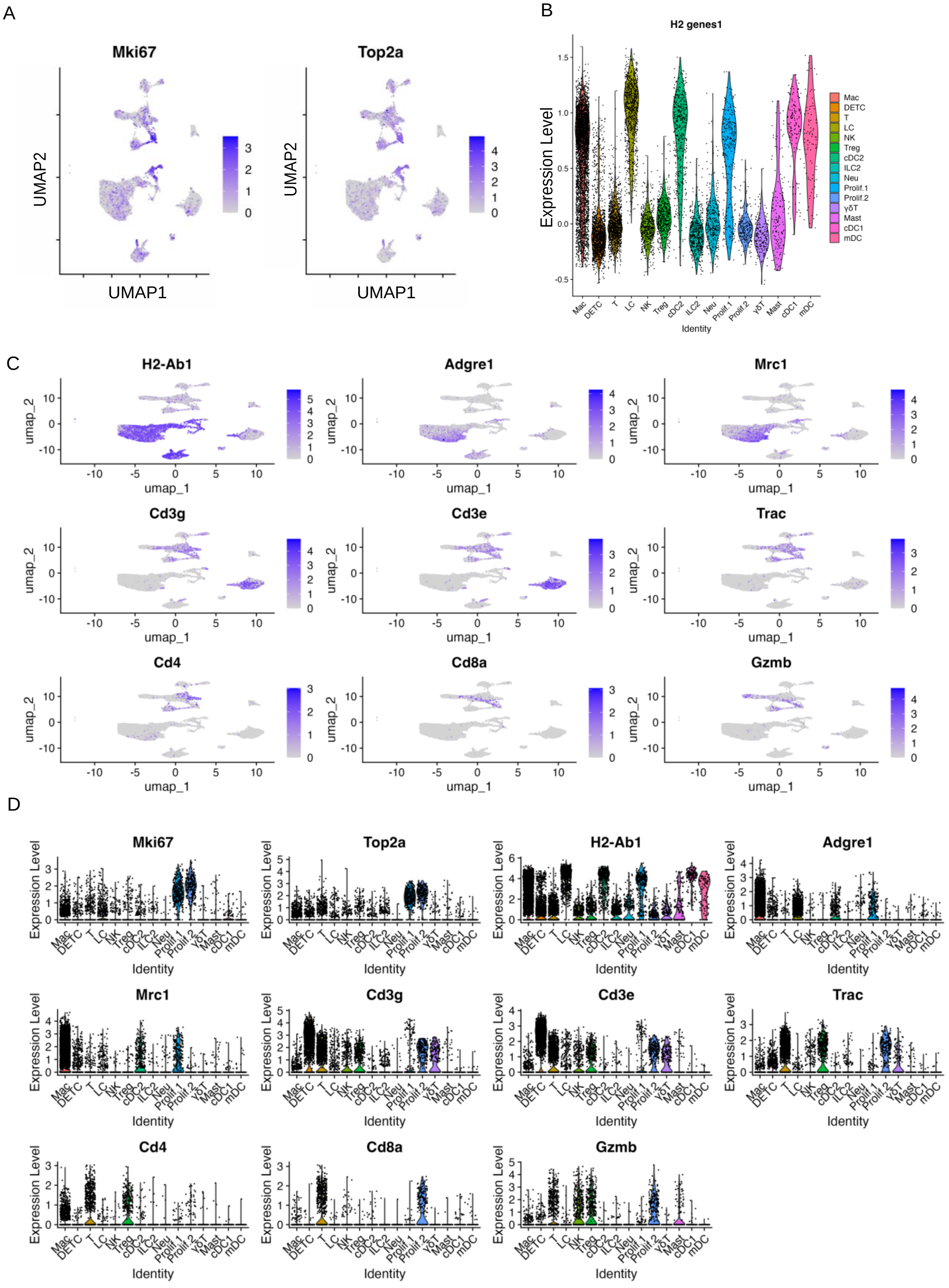

Figure S2. Two proliferating cell clusters. (A) Feature plots showing two proliferative marker genes Ki67 and Top2a selectively expressed in two proliferating cell clusters. (B) Expression of MHC genes across immune cell clusters identifying APC cells such as Mac, LC, cDC1, cDC2, and mDC. (C) Feature plots showing the expression of H2-Ab1, Adgre1, Mrc1, Cd3g, Cd3e, Trac, Cd4, Cd8a, and

Gzmb across immune cell clusters. (D) Violin plots of showing the expression of Mki67, Top2a, H2-Ab1, Adgre1, Mrc1, Cd3g, Cd3e, Trac, Cd4, Cd8a, and Gzmb across immune cell clusters.

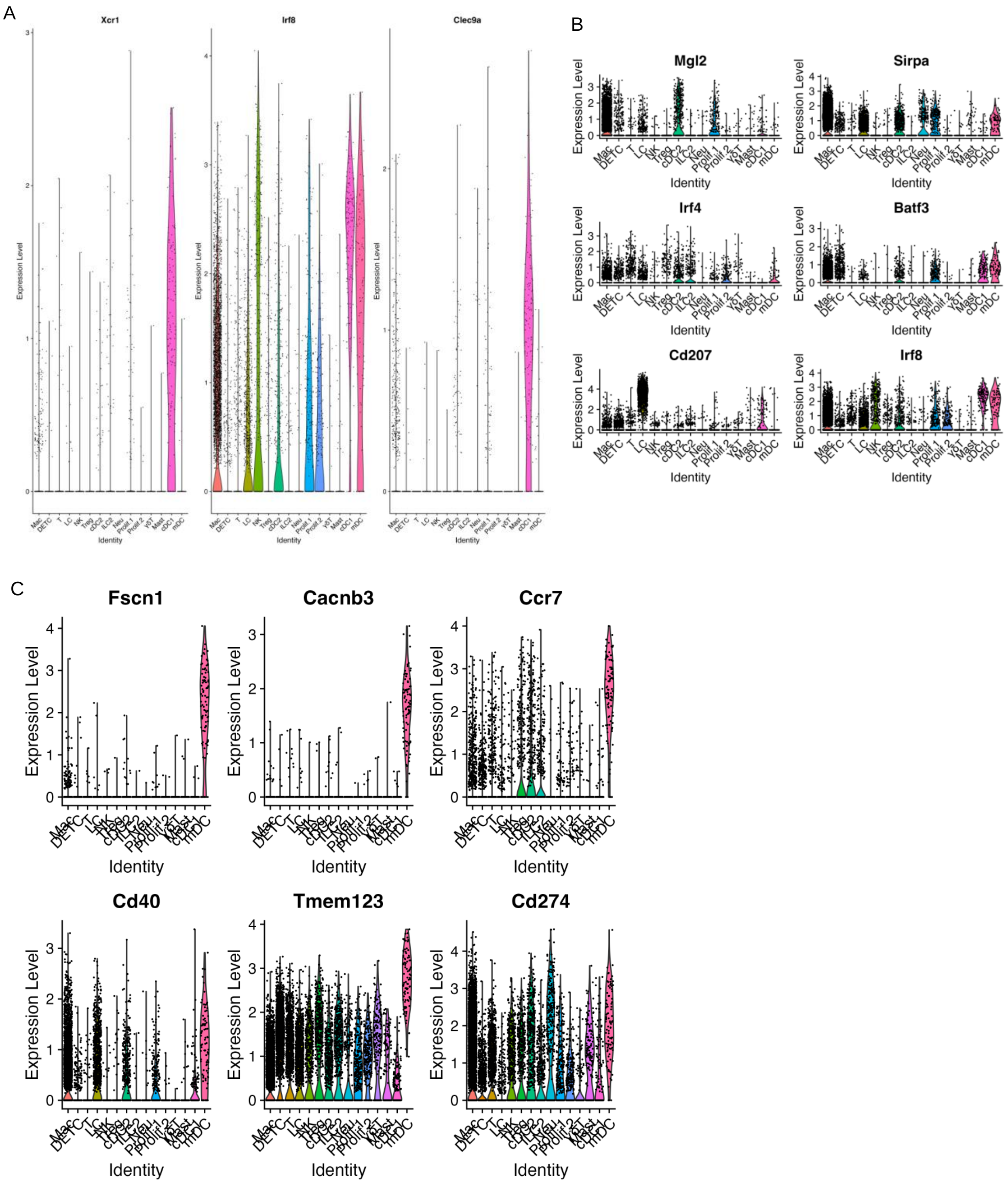

Figure S3. DC marker genes across immune cell clusters. The expression of cDC1 marker genes Xcr1, Irf8, and Clec9a across immune cell clusters. (B) Violin plots showing the expression of Mgl2, Sirpa, Irf4, Batf3, Cd207, and Irf8 across immune cell clusters. (C) Expression of mDC marker genes including Fscn1, Cacnb3, Ccr7, Cd40, Tmem123, and Cd274 across immune cell clusters.

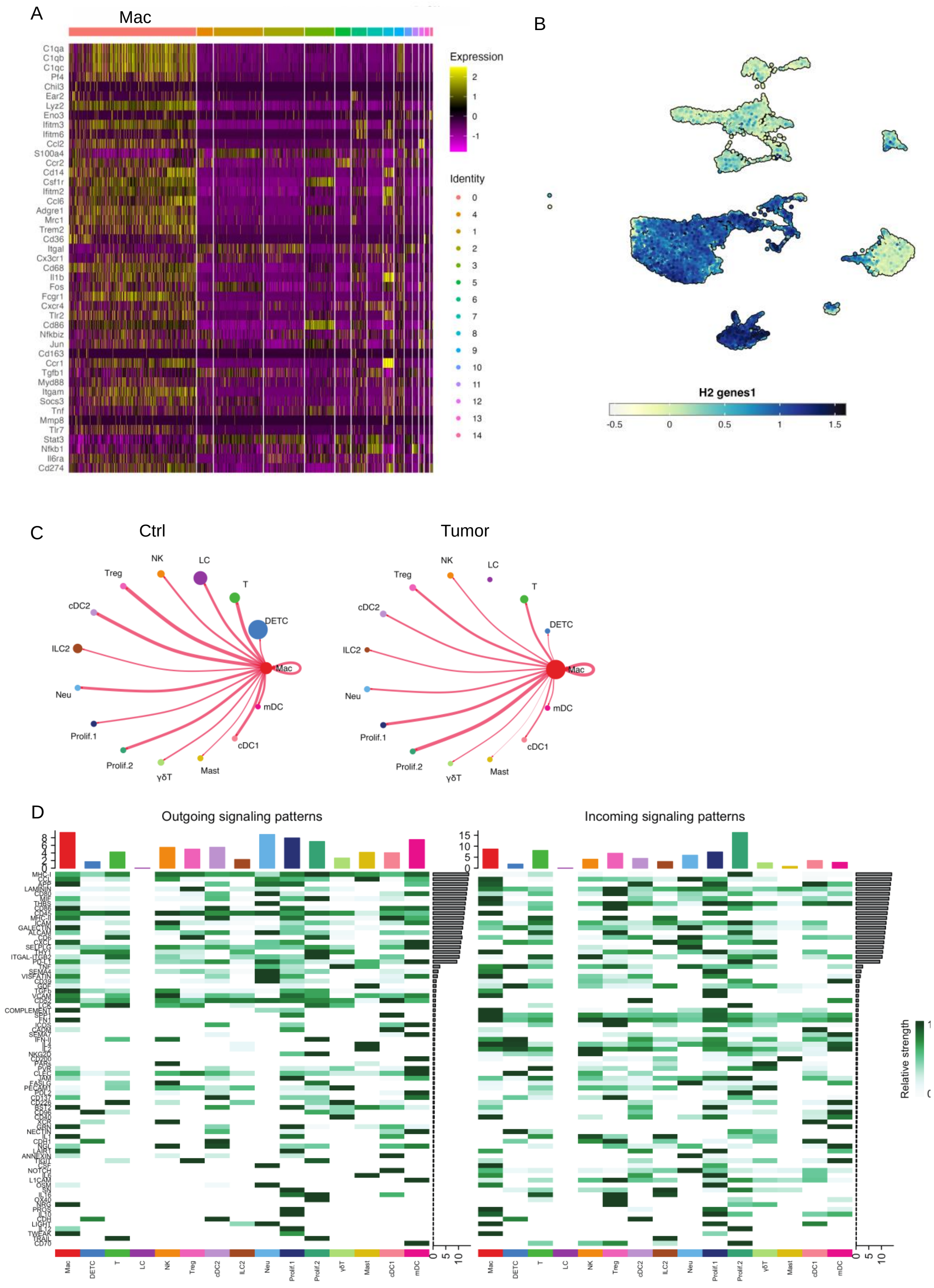

Figure S4. Macrophage markers. (A) A heatmap of macrophage markers showing the macrophage cell cluster. (B) Feature plot showing the H2 genes of APC clusters. (C) The circle diagram illustrates the signal crosstalk between macrophages and other immune cells, with the thickness representing the signal strength. (D) CellChat heatmap showing the relative strength of outgoing and incoming signaling pathways for each cell type. Vertical bars on top of the heatmap indicate overall

importance of the cluster in the respective signaling pattern (i.e. outgoing or incoming). Horizonal bars indicate overall importance of the signaling pathway in the respective signaling pattern.

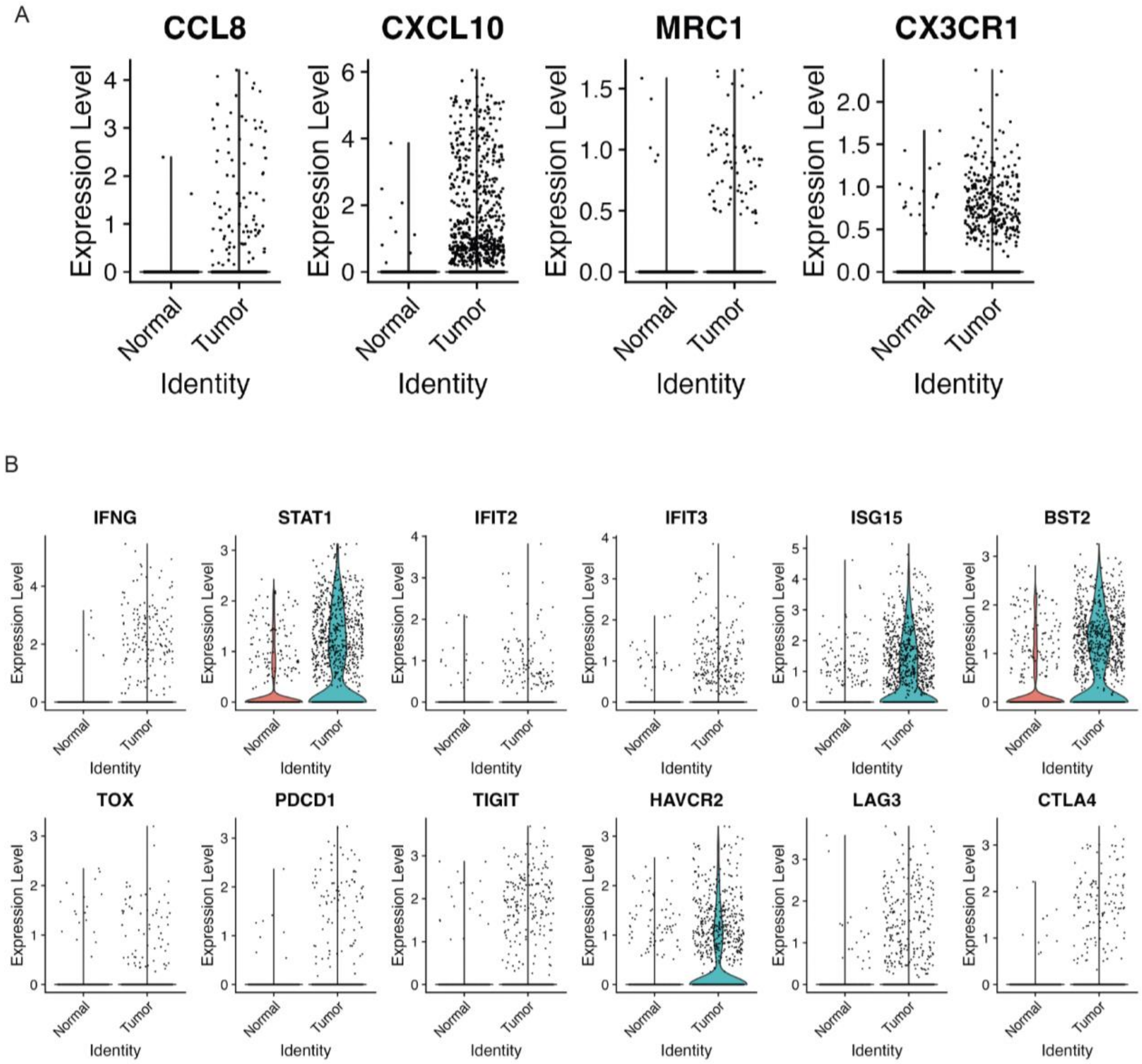

Figure S5. Gene expression analysis in human cSCC patients using the publicly available dataset GSE144236. (A) Expression level of CCL8, CXCL10, MRC1, and CX3CR1 in the macrophage population in normal skins and human cSCC tumor patients. (B) Expression level of interferon pathway genes including IFNG, STAT1, IFIT2, IFIT3, ISG15, and BST2, and exhaustion marker genes including TOX, PDCD1, TIGIT, HAVCR2, LAG3, and CTLA4 in the T cell population in normal skins and human cSCC tumor patients.

Table S1. scRNA-seq dataset metrics.

Samples	Estimated Number of Cells	Post-filtering number of Cells	Mean Reads per Cell
C57_1	3499	2898	87,090
C57_2	3862	3048	81,098
2323_Tumor_1	5176	4748	45,977
2323_Tumor_2	2258	1989	100,120

Table S2. Representative marker genes for immune cell populations (related to Figure 1 and Figure 2).

	classic makers	% cell in cluster	Adjusted P-value	Extended markers	% cell in cluster	Adjusted P-value	Negative markers
Macrophage	Adgre1	69.6	0	Mrc1	70.6	0	
	Ms4a7	51	0	Folr2	19	6.01×10^{-189}	
	C1qc	69.5	0	Arg1	12.6	2.15×10^{-148}	
	Itgam	67.8	0				
	Cd68	77.8	0				
Type 2 conventional dendritic cells (cDC2)	H2-Ab1	98	2.13×10^{-85}				
	H2-Aa	96.2	2.60×10^{-78}				
	Cd209a	63.7	0				
	Cd209d	30.6	7.32×10^{-224}				
	Irf4	37.9	7.19×10^{-37}				
	Mgl2	53.2	5.04×10^{-72}				
Type 1 conventional dendritic cells (cDC1)	H2-Ab1	100	1.76×10^{-28}				
	H2-Eb1	100	4.84×10^{-33}				
	Clec9a	82.8	0				
	Xcr1	78.6	0				
	Irf8	94.5	1.29×10^{-73}				
	Batf3	67.6	5.67×10^{-43}				
mature/migratory Dendritic cells (mDC)	Fscn1	87.1	0				
	Cancnb3	95.7	0				
	Ccr7	95.7	4.87×10^{-136}				
	Cd40	82.8	5.24×10^{-34}				
	Tmem123	100	3.30×10^{-53}				
	Cd274	96.8	2.03×10^{-20}				
Langerhans cells (LC)	H2-M2	95.4	0				
	Cd207	98.3	0				
	Epcam	85	0				
	Cd24a	71.5	0				
	Csf1	88.8	7.10×10^{-214}				
Dendritic Epidermal T cells (DETC)	Cd3e	92.6	0				Cd4
	Cd3g	86.4	0				Cd8a
	Trdc	95.5	0				
	Tcrg-C1	82.3	0				
	Thy1	76.9	0				
Dermal gamma delta T cells	Cd3e	92.6	0				Cd4
	Cd3g	86.4	0				Cd8a
	Tcrg-C1	82.3	0				
	Trdc	95.5	0				
	Thy1	76.9	0				
Natural Killer Cells (NK)	Gzma	33.4	0				
	Klra7	40.5	0				
	Eomes	42.6	0				
	Ncr1	40.3	0				
	Prf1	55.9	3.70×10^{-140}				
	Klra8	27.3	0				
	Klrb1c	30.7	0				
Neutrophils (Neu)	S100a8	96.7	0				
	S100a9	97.6	0				
	Csf3r	92.4	1.44×10^{-297}				
	Spi1	67.8	4.03×10^{-18}				
	Slc11a1	76	6.08×10^{-117}				

Mast Cells	Gata2	89.9	0	Mctp4	98	0
	Ms4a2	62.4	0	Kit	88.6	2.93×10^{-205}
Type 2 innate lymphoid cells (ILC2)	Gata3	86.7	0			Cd3d/e/g
	Rora	99.6	0			Eomes
	Il7r	90	3.52×10^{-207}			Rorc
	Il5	48.1	0			Tbx21
	Il13	41.1	$5.79\times10-^{206}$			Cd4
Treg	Foxp3	63.1	0			
	Cd4	42.8	0			
	Tnfrsf18	56.8	7.95×10^{-109}			
	Ctla4	92.6	0			
Prolif.1	Mki67	96.8	0			
	Top2a	96.1	0			
	H2-Ab1	91	4.77×10^{-08}			
	H2-Aa	92	5.77×10^{-09}			
	Adgre1	51.8	0.02			
	Mrc1	56.6	1.32×10^{-10}			
Prolif.2	Mki67	97.9	8.58×10^{-293}			
	Top2a	96.6	3.23×10^{-284}			
	Cd3g	82.9	2.49×10^{-51}			
	Cd3e	81.2	5.03×10^{-41}			
	Trac	80.8	1.07×10^{-140}			
	Cd4	20.1	0.02			
	Cd8a	58.1	5.37×10^{-225}			
	Gzmb	65	8.50×10^{-150}			
T cells	Cd3d	57.6	1.60×10^{-130}			
	Trac	62.8	0			
	Trbc1	45.4	2.15×10^{-61}			
	Cd4	29.1	5.92×10^{-135}			
	Cd8a	33	0			
	Cd8b1	32.2	0			
